# Clinical trialist perspectives on the ethics of adaptive clinical trials: a mixed-methods analysis

**DOI:** 10.1186/s12910-015-0022-z

**Published:** 2015-05-03

**Authors:** Laurie J Legocki, William J Meurer, Shirley Frederiksen, Roger J Lewis, Valerie L Durkalski, Donald A Berry, William G Barsan, Michael D Fetters

**Affiliations:** Department of Family Medicine, University of Michigan, 1018 Fuller Street, Ann Arbor, MI 48109 USA; Departments of Emergency Medicine and Neurology, University of Michigan, Ann Arbor, MI USA; Department of Emergency Medicine, University of Michigan, Ann Arbor, MI USA; Department of Emergency Medicine, Harbor-UCLA Medical Center, Los Angeles, CA USA; Division of Public Health Sciences, Medical University of South Carolina, Charleston, SC USA; Department of Biostatistics, University of Texas M.D. Anderson Cancer Center, Houston, TX USA; Los Angeles Biomedical Research Institute, David Geffen School of Medicine, University of California Los Angeles, Los Angeles, CA USA; Berry Consultants, Austin, TX USA

**Keywords:** Adaptive clinical trials, Mixed methods, Ethics, Visual analogue scale, Clinical trials, Qualitative research, Emergency medicine, Neurological emergency, Informed consent, Equipoise

## Abstract

**Background:**

In an adaptive clinical trial (ACT), key trial characteristics may be altered during the course of the trial according to predefined rules in response to information that accumulates within the trial itself. In addition to having distinguishing scientific features, adaptive trials also may involve ethical considerations that differ from more traditional randomized trials. Better understanding of clinical trial experts’ views about the ethical aspects of adaptive designs could assist those planning ACTs. Our aim was to elucidate the opinions of clinical trial experts regarding their beliefs about ethical aspects of ACTs.

**Methods:**

We used a convergent, mixed-methods design employing a 22-item ACTs beliefs survey with visual analog scales and open-ended questions and mini-focus groups. We developed a coding scheme to conduct thematic searches of textual data, depicted responses to visual analog scales on box-plot diagrams, and integrated findings thematically. Fifty-three clinical trial experts from four constituent groups participated: academic biostatisticians (*n* = 5); consultant biostatisticians (*n* = 6); academic clinicians (*n* = 22); and other stakeholders including patient advocacy, National Institutes of Health, and U.S. Food and Drug Administration representatives (*n* = 20).

**Results:**

The respondents recognized potential ethical benefits of ACTs, including a higher probability of receiving an effective intervention for participants, optimizing resource utilization, and accelerating treatment discovery. Ethical challenges voiced include developing procedures so trial participants can make informed decisions about taking part in ACTs and plausible, though unlikely risks of research personnel altering enrollment patterns.

**Conclusions:**

Clinical trial experts recognize ethical advantages but also pose potential ethical challenges of ACTs. The four constituencies differ in their weighing of ACT ethical considerations based on their professional vantage points. These data suggest further discussion about the ethics of ACTs is needed to facilitate ACT planning, design and conduct, and ultimately better allow planners to weigh ethical implications of competing trial designs.

**Electronic supplementary material:**

The online version of this article (doi:10.1186/s12910-015-0022-z) contains supplementary material, which is available to authorized users.

## Background

Fixed randomized clinical trials (RCTs) are widely considered the gold standard for determining the relative efficacy of medical treatments [1]. However, during the design of confirmatory trials, even after early phase exploratory trials have been completed, there is often substantial uncertainty regarding how best to administer the experimental treatment to maximize the likelihood of benefit. For example, the optimal dose of the experimental medication, the best duration of therapy, or the population most likely to benefit, may all remain unclear. This situation creates uncertainty as to the optimal parameters for the proposed trial, yet under the fixed approach to trial design and conduct, all key trial parameters must be defined before patient enrollment and then held constant [2].

Adaptive clinical trials (ACTs) represent an innovative approach to trial design and conduct [3], where the primary goal of adaptations is to improve scientific value and statistical efficiency. ACTs are designed to take advantage of accumulating information by allowing key trial parameters to be modified in response to accumulating data according to predefined rules [4-8]. As in all confirmatory trials that lead to practice change or regulatory approval, well-designed ACTs follow rigid requirements and are carefully designed to control the risk of error including type I and type II error rates [4]. Adaptive designs may incorporate a broad spectrum of potential changes to trial conduct, with early trial termination rules based on statistical boundaries being one of the simplest adaptations. Most large clinical trials include stopping boundaries for benefit, futility, or harm; these provide quantitative guidance to safety monitoring committees [9]. On the other hand, more complex adaptive trials can be designed to identify the most promising treatments for specific subpopulations within a disease and tailor trial enrollment and randomization to maximize the information gained [10]. The advantages and disadvantages of some types of adaptations are better understood than others. While adaptive designs have existed for some time, they are receiving renewed emphasis and funders and oversight bodies have suggested expanded use [11]. Despite the growing interest, few adaptive designs have been used relative to the overall number of trials conducted. This is especially true in confirmatory phase trials. In addition, few clinical trialists have actual experience with adaptive designs and direct knowledge of ACTs.

One prerequisite of conducting any clinical trial is the careful consideration of the ethics of the trial procedures [12]. Trial design impacts scientific ethics (it is unethical to conduct an invalid study), individual ethics (which patients are allowed to participate and what scientific and experimental procedures are they exposed to) and collective ethics (the need to discover better treatments or treatments for untreatable conditions to improve future patient outcomes) [2]. The ethical aspects of fixed RCTs and ACTs differ. Pullman characterizes the difference as fixed RCTs favoring collective ethics and ACTs favoring individual ethics [13]. The statistical focus of fixed RCTs, particularly those studies designed to change clinical practice or lead to drug or device approval (phase III or confirmatory), is to test hypotheses about treatment effect. In such trials, the primary goal of a trial is to improve the treatment for the broader community, with individuals within the trial only conferring benefit if they are randomly allocated to the better treatment (if one is indeed found to be better). Through informed consent, potential research participants decide themselves whether to be in a trial, or opt for conventional therapy [13].

ACTs have different ethical nuances than more traditional, fixed RCTs [14]. In addition to the need for an informed decision whether to participate in the trial or choose conventional therapy, there are other ethical considerations. For example, some adaptive design strategies increase the probability of receiving the more effective treatment while maintaining the scientific rigor of the trial [10,15]. This emphasizes individual ethics because the objective is to treat as many patients effectively as is possible [13]. The potential advantage over fixed RCTs is that more patients will be assigned to the better-performing treatment arm(s), regardless of which arm(s) that turns out to be. This introduces one example of a potentially favorable ethical outcome not present in a fixed RCT. A more recent review focused on the potential ethical risks of such trial designs, particularly highlighting the lack of equipoise that develops as the trial goes on and the inherent injustice that later enrolling subjects will get better treatments than earlier enrolling subjects [16]. A recent article induced a healthy debate regarding the ethics of one specific form of adaptive trials: those utilizing outcome (also known as response) adaptive randomization [17-22]. Clinical trials in the critically ill are particularly challenging [23]. Interesting, a hypothetical clinical trial scenario demonstrated greater participation when participants were offered a trial with response adaptive randomization [24].

The ADAPT-IT project is an initiative funded by the U.S. National Institutes of Health (NIH) and the Food and Drug Administration (FDA). The first aim of the study is to develop five confirmatory, ACT designs evaluating treatments for neurological emergencies [10]. For the ACT trial design work, the research team set out to develop clinical trials focusing primarily on two broad categories of adaptation, namely, frequent interim analysis and response-adaptive randomization. Selected features of these important adaptations are illustrated in Table 1.

**Table 1:** **Key features and approaches to adaptations and ethical considerations of frequent interim analysis and response-adaptive randomization for adaptive trial designs**

**Trial element**	**Key features**	**How trial is adjusted**	**Comment**	**Ethical considerations**
**Frequent interim analysis (FIA)**	• Trial success and futility are repeatedly assessed.	• Terminate when successful	• Lower total trial size when treatment is more effective than anticipated or has little or no effect	• Ethical advantage accrues as fewer participants may be required to terminate a successful trial or negative trial.
				• Provides an ethical advantage for the population of patients involved by exposing fewer participants to ineffective treatments.
		• Terminate if continuation is unlikely to succeed		
	• Assess:			
				• If more participants are needed when experimental treatment appears to have marginal effectiveness, the probability of benefit will likely exceed 50:50; this potentially poses an ethical advantage for the population of participants because the chance of getting an effective treatment, even if marginal, may be greater than in a trial without FIA
		• May increase sample size per prespecified rules		
			• May result in a larger required sample size if experimental therapy has marginal effectiveness	
	− evidence of treatment efficacy			
				• Offers ethical advantage by avoiding erroneous conclusion that a marginally effective treatment is ineffective, and obfuscating use for individuals who could benefit from treatment
	− rates of adverse events			
	− patient safety			
**Response-adaptive randomization (RAR)**	• Randomization proportions are varied during the course of the trial.	• Reallocation between arms to increase the fraction of participants who receive the most effective treatment	• Can decrease the required sample size by allocating patients in arms most likely to be efficacious	• Decreasing the sample size offers ethical advantage to the population of eligible participants by exposing fewer of them to ineffective treatments.
				• Ethical benefits are more likely to accrue for the population of patients in the trial as more than half are likely to be enrolled in arms with probability of benefit
	• Ongoing modification			
				• Ethical benefit accrues over the course of the trial for individual participants, who will be more likely to receive effective treatment.
	• Allocate patients to different treatment arms			
				• Efficiency enhances ethical benefits for society by minimizing unnecessary costs and allowing precious resources to be re-allocated to other research for other effective treatments.
			• As in randomized, controlled trials without RAR, participants enrolled early have no advantage for being in an arm with the more effective treatment, but enrollment later enhances probability of enrollment in the effective treatment arm	
				Offers ethical advantage to the population of persons in society who need the treatment under evaluation, since the trial results of an effective treatment will be known sooner, providing clinicians and patients with treatments known to offer benefit
	• Allocate patients to different doses of an active agent			
		• May improve the statistical efficiency of the trial if there are more than two arms		

The second aim of the project is to study the ACT design process itself including the interactions and views of the constituent clinical design experts [10]. The participating trial experts include clinician researchers, consultant biostatisticians who specialize in RCTs, academic statisticians who specialize in complex, multicenter RCTs, regulators from the FDA and NIH as well as patient advocates (Table 2). Given the complete lack of empirical literature on clinical trial experts’ views on the ethical aspects of ACTs, the breadth and diversity among participants in the design process, and the need for them to work together, this project provided a unique opportunity to explore clinical trial experts’ views about the ethical features of ACTs. A better understanding of their views on ethical features of ACTs could advance the discussion about ethical aspects of adaptive designs, identify any areas of contention, promote dialogue to understand benefits and limitations of ACTs, and facilitate acceptance of ACTs by the broader research community.

**Table 2:** **Characteristics of clinical trial experts**

**Characteristic**	**Academic biostatisticians**	**Consultant biostatisticians**	**Academic clinicians**	**Other stakeholders**
Mean age	47	45	49	52
Female, *21/53 (39.6%)*	3 (60)	1 (17)	5 (23)	12 (60)
Highest degree, *53*				
MD or equivalent	0	2 (33)	20 (91)	7 (35)
PhD	5 (100)	4 (67)	4 (18)	12 (60)
Primary work location, *52*				
University/University hospital	5 (100)	1 (17)	21 (96)	3 (15)
Community hospital	0	0	0	0
Government (NIH or FDA)	0	0	0	15 (75)
Consulting firm		4 (67)	1 (5)	
Other	0	0	0	2 (10)

Hence, the purpose of this study was to elucidate the opinions of clinical trial experts regarding their beliefs about ethical aspects of ACTs. Participants were asked to consider ethical advantages and potential ethical disadvantages from the perspective of patients, researchers and society.

## Methods

### Study design

We conducted a convergent mixed-methods design that used a 22-item ACT beliefs survey with visual analog scales (VAS), free-text survey responses, and mini-focus groups, a type of group interview [25,26]. The VAS instrument and the mini-focus group discussion guides are available as Additional file 1 and Additional file 2. A mixed methods approach was implemented to elucidate participants’ beliefs, to identify the reasoning behind the beliefs expressed, and to integrate the data together to provide the broadest possible understanding. Study instruments were developed by an ethicist (MF) and experts in both traditional and adaptive clinical trials (WM, RL), and focused on the evaluation of ethical advantages and disadvantages of adaptive clinical trial designs. Data were collected with self-administered surveys, either by paper or on the Web, by using VAS and free-text responses. Reminders were sent to individuals who had not completed the surveys and frequent announcements were made during trial planning meetings encouraging subjects to Data also were collected during five mini-focus groups with four to six clinical trial experts per group [25,26]. The mini-focus group guide was specifically designed with topics to parallel the items on the VAS instrument so that results from both instruments could be mapped together. The University of Michigan human subjects review committee deemed this project exempt from Institutional Review Board oversight per United States federal regulations (45 CFR 46.101(b). Participants received information about the study and the research intent prior to data collection.

### Settings and participants

Participants were recruited as part of an ongoing NIH-FDA–funded research project exploring the incorporation of ACT designs into an existing neurological emergencies treatment trials network (NETT) [27]. Project investigators held a series of meetings that included experts in ACT design and investigators interested in developing an ACT for specific research topics related to neurological emergencies. A mixed methods team assessed the ACT development process during these meetings and conducted the analysis. Data were collected between January and August of 2011. Participants were classified as belonging in one of the following groups of clinical trial experts: academic biostatisticians from NIH-funded clinical trial networks (*n* = 5) with substantial experience running phase III trials, consultant biostatisticians working in academic or industry settings with specific experience in Bayesian adaptive designs (*n* = 6), academic clinicians (*n* = 22), and other stakeholders, e.g., NIH officials, FDA statisticians, medical officers, and patient advocates — all experts in the planning of clinical trials (*n* = 20). Instead we asked those surveyed for their opinions about how patients might view the advantages and disadvantages of ACTs.

### Variables

Survey and mini-focus group questions were formulated to gather opinions of the clinical trial experts regarding the ethical advantages and disadvantages of ACT designs. Participants considered advantages and disadvantages from the perspectives of the patient, the researcher, and society as a whole.

### Data sources

Mini-focus groups [25,26] with clinical trial experts were conducted before the initial face-to-face meetings for four of the five trials. These mini-focus group sessions were digitally recorded and transcribed verbatim, and the data were entered into Atlas.ti v6.0 [28] for data management.

Participants answered the VAS items by completing a paper survey or a Web-based survey. The VAS allowed participants to mark a point of agreement on a continuum ranging from “definitely not”, to “probably not”, to “possibly”, to “probably”, to “definitely”. We used a 100-point scale to allow greater flexibility in examining differences than a five-point structured Likert scale would allow. To compute a quantitative measure of a participant’s assessment, we assigned the lowest anchor a value of 0 and the highest anchor a value of 100 and calculated a level of agreement score based on the point chosen by the participants for the VAS items.

### Data analysis

Descriptive statistics (proportions and means) were calculated for demographic variables. The VAS data were depicted by using box plots visually illustrating the median, interquartile range, 95% confidence interval, and outliers for each survey question. Qualitative data from the five mini-focus groups and free-text responses from the surveys were analyzed independently by two investigators (LL, SF) from the mixed-methods evaluation team with an 88% intercoder agreement [29]. The development of a coding scheme was based initially on the thematic basis of the interview guide and revised to reflect the primary themes that emerged from the analysis. The VAS scores using box-plot diagrams were integrated with comments by constituency groups for sub-analyses to merge quantitative ratings with qualitative textual data and with representative quotations [30]. As a “member check”, the process of allowing participants in qualitative research to reflect on the findings and provide the investigators feedback on the extent to which the findings represent their viewpoints, a near-final draft of this manuscript was sent to a purposively, chosen member from each of the four constituency groups—the academic biostatisticians, the consultant biostatisticians, the academic clinicians, and the “other” stakeholders group. These group members responded with suggestions for wording changes that were integrated into the manuscript.

### Demographic characteristics of the sample

The survey was offered to 64 participants in the ADAPT-IT project, of whom 53 individuals participated from four constituent groups: academic biostatisticians (*n* = 5); consultant biostatisticians (*n* = 6); academic clinicians (*n* = 22); and other stakeholders, including FDA and NIH personnel and patient advocates (*n* = 20). The mean age (± standard deviation) of participants was 49.0 ± 10.9 years. The majority were men (60%) who were physicians (55%) working in a university hospital setting (57%). Approximately ten individuals who were participants in the survey procedures were also co-investigators on the ADAPT-IT project; these individuals were not directly involved in the data collection or analysis of the textual and survey data; several were involved in the criticial revision and interpretation presented in this manuscript.

## Results

**Figure 1:**
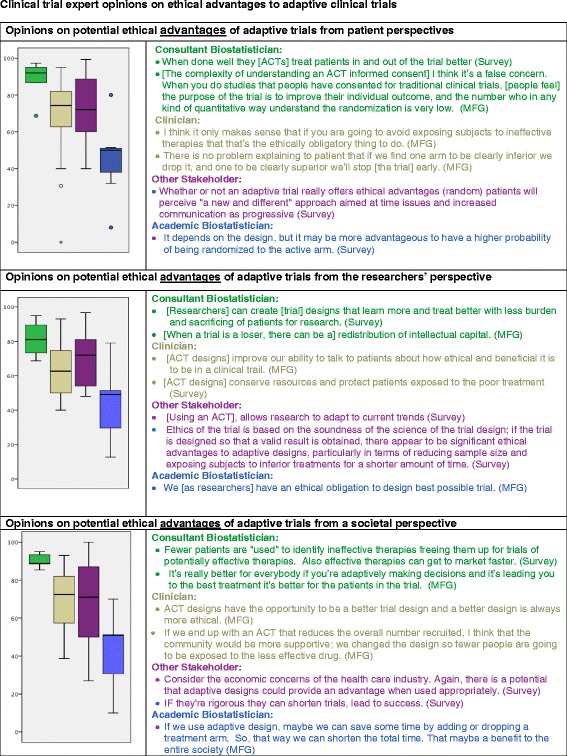
Clinical trial expert opinions on ethical advantages to adaptive clinical trials from the patient, researchers, and societal perspectives. The above Likert scales anchor from 100 (definitely) to 0 (definitely not). Boxplots represent median and inter-quartile range. Responses are colored by respondent type as are boxplots.

**Figure 2:**
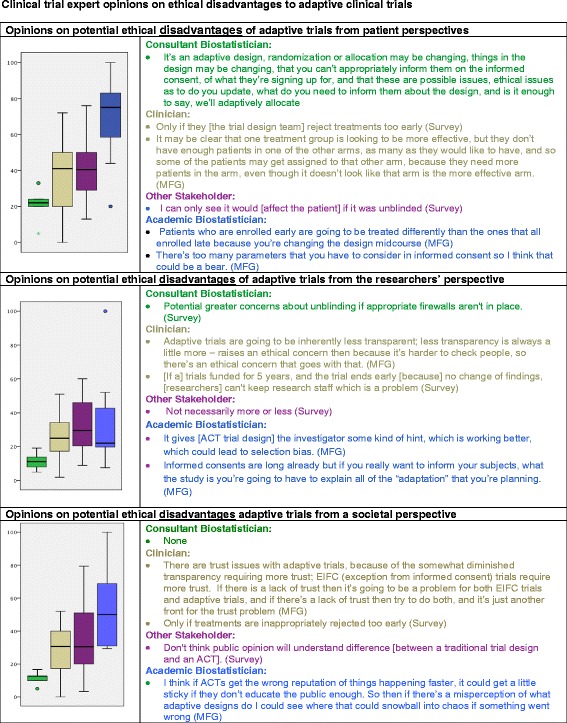
Clinical trial expert opinions on ethical disadvantages to adaptive clinical trials from the patient, researchers, and societal perspectives. The above Likert scales anchor from 100 (definitely) to 0 (definitely not). Boxplots represent median and inter-quartile range. Responses are colored by respondent type as are boxplots.

Figures 1 and 2 illustrate stakeholders’ opinions of the ethical advantages and potential disadvantages of ACTs when considered from the patient, researcher, and societal perspectives. The section on the left side of each figure provides the participants’ ratings of the ethical advantages on the VAS, with the lowest anchor at 0 to signify definitely not agreeing with the statement and the highest anchor at 100 to signify definite agreement with the statement. In addition, the right side of each figure features illustrative opinions from the free-text response on the survey and the mini-focus groups.

### Potential ethical advantages of ACTs

#### Opinions regarding the patient perspective

The consultant biostatisticians, academic clinicians, and other stakeholders rated the effects of ACTs on patients as definitely advantageous to probably advantageous, whereas academic biostatisticians’ responses ranged from possibly having to probably not having ethical advantages (Figure 1). Participants suggested that the greatest advantage from the patient’s perspective focused on the potential benefit to the study patient. A member of the “other” stakeholder group offered an illustrative response regarding the favorable benefit: “*Response-adaptive randomization gives patients a better chance of being randomized [to the better arm in a clinical trial]*”. Another participant, a clinician, raised the point that ACTs will not expose more people than necessary to an ineffective drug: “*[The] main ethical advantage is that you potentially reduce the number of patients who get the ineffective drug, and that actually is a plus*”. Consultant biostatisticians highlighted the importance of understanding the individual ethics of a trial:

“*The adaptive approach, when properly done, pays more attention to individual ethics, in the sense that it ensures that the trial doesn’t continue when there isn’t a chance that either therapy is effective or that the therapy is more effective than the control. It can be designed to improve the outcomes of patients within the study*”.

Academic biostatisticians also thought there was a potential benefit to response-adaptive randomization, as one opined, “*The idea of response-adaptive randomization is to let later patients have a higher likelihood of getting a drug that seems to be working better*”. One patient advocate suggested that the advantage to an ACT is that it is a new approach,

“*Whether or not an adaptive trial really offers ethical advantages, patients will perceive "a new and different" approach aimed at time issues and increased communications as progressive”.*

### Opinions regarding the researcher perspective

Three groups assessed the ethical advantages from researchers’ perspectives positively, with ratings in the range of definitely to probably among consultant biostatisticians and probably to possibly among the academic clinicians and other stakeholders (Figure 1). One clinician noted that ACT designs:

*“make me feel like it improved our [*clinician’s*] ability to talk to the patient about how ethical and beneficial it is to be in clinical trials. So, again, assuming that we’re talking about pre-specified changes, I think it would - there would be substantial ethical issues if we told the patients we’re going to put you in the trial, and we may change the eligibility criteria; we may change the outcome criteria; we may change everything; we have no idea where we’re going with this. I don’t think that that is a fair thing to put a patient into. But if we’re talking about adaptive clinical trials where we are pre-specifying what our adaptations are, I think that that’s a very beneficial thing to patient care”.*

From the perspective of researchers and also society in this case, participants also pointed to other advantages of ACTs versus a more traditional RCT without reallocation, including, as noted by a consultant biostatistician,

“there’s the intellectual capital that can get wasted. If all of your statisticians and your study people are no longer focusing on this trial that’s a loser, they can be working on something else. So I think that the overall efficiency of the medical research enterprise is something that ought to be important to all of us in the adaptive design part”.

Academic biostatisticians rated the ethical advantages to be in the range of possibly advantageous to probably not advantageous.

### Opinions from a societal perspective

Regarding opinions from a societal perspective, the consultant biostatisticians all indicated definite ethical advantages. Responses from the academic clinicians and other stakeholders ranged from probably to possibly offering advantages, and the academic biostatisticians from possibly offering advantages to probably not offering advantages (Figures 1 and 2). Some participants identified specific advantages from the societal perspective, such as, “[ACTs] answer [questions] at less societal cost” (other stakeholder). One consultant biostatistician suggested:

“*fewer patients are "used" to identify ineffective therapies freeing them up for trials of potentially effective therapies. Also effective therapies can get to market faster. Plus were more likely to have a better dose make it to market.*,” (consultant biostatistician).

An academic biostatisticians opined:

“*If we use adaptive design, maybe we can save some time by adding or dropping a treatment arm. So, that way we can shorten the total time. That maybe a benefit to the entire society [for the patient or the trial], I don’t think it’s ethical either good or bad thing. It could be either”.*

### Potential ethical disadvantages of ACTs

#### Opinions regarding the patient perspective

More than the other groups, academic biostatisticians assessed the ethical disadvantages of ACTs for patients to be high; their VAS responses ranged from probable disadvantages to definite disadvantages. Citing time of enrollment, one academic biostatistician stated, “*Patients who are enrolled early are going to be treated differently than the ones* … [who are] *enrolled late, because you’re changing the design midcourse*”. The academic biostatisticians also expressed concerns about informed consent:

*“I think it will be a mess, the informed consents are long already but if you really want to inform your subjects, potential subjects, what the study is you’re going to have to explain all of that adaptations that you’re planning. So then to explain how your chances are going to change during the course of the study, it definitely could complicate”.*

The clinician group and the “other” stakeholder group assessed potential disadvantages with lower scores; they generally perceived that patients would probably have no disadvantages, unless “*treatments were rejected too early*” (clinician) or “*if the process was unblinded*” (other stakeholder group). Consultant biostatisticians indicated that ACTs had no definite disadvantages to patients, however one acknowledged that informed consent was a complicated issue:

*“an adaptive design, randomization or allocation may be changing, things in the design may be changing, that you can’t appropriately inform them* [patients] *on the informed consent, of what they’re signing up for, and that these are possible issues, ethical issues as to do you update, what do you need to inform them about the design, and is it enough to say, we’ll adaptively allocate. We don’t change the informed consent when the randomization changes. We tell them that this will be adapted in the following way. They may not understand it, but which patient does understand the protocol and be informed, is truly informed*”.

### Opinions regarding the researcher perspective

All four constituent groups rated adaptive trials as having few potential ethical disadvantages when considered from the researcher’s perspective (Figure 2). Ratings from the researcher’s perspective ranged from possibly to probably not among other stakeholders, probably not among the academic clinicians and academic biostatisticians, and definitely not among the consultant biostatisticians. In mini-focus group discussions among academic biostatisticians, concern was raised about the risks of unintentional unblinding that could occur and how this could lead to selection bias from academic clinicians who might then consciously or unconsciously steer particular patients away from enrolling in the trial. Noted one such participant,

“[ACT design] *gives the investigator some kind of hint* [regarding] *which* [treatment] *is working better* [and] *which could lead to selection bias because now one is better than the other side. The investigator may purposely enroll or reject a potential subject for or against a specific arm* (academic biostatistician)”.

Consultant biostatisticians acknowledged that unblinding might be an issue, especially if “*appropriate firewalls are not in place*” to prevent it. Clinician stakeholders thought that ending a trial early could be a disadvantage if doing so led to a loss of funding, resulting in an inability to “keep research staff, which is a problem”. This sentiment illustrates a potential conflict of interest for a funded investigator, as an ACT conducted with an early stopping rule could put an investigator at a financial disadvantage if grant funding ends earlier than anticipated. The “other” stakeholder group did not indicate any disadvantages from the researcher perspective.

### Opinions from a societal perspective

Three of the constituent groups—the academic clinicians, other stakeholders, and academic biostatisticians—rated disadvantages of ACTs from a societal perspective in a range of possibly to probably not disadvantageous, whereas the consultant biostatisticians rated ACTs as definitely not having ethical disadvantages from this perspective (Figure 2). One clinician commented that the only disadvantage would be if “*treatments were inappropriately rejected to early”.* A member of the “other” stakeholder group “*didn’t think that public opinion would understand the difference*” between an adaptive and a traditional design.

### Trends according to intragroup and intergroup variations across all ethical questions

Across all the ethical domains provided (Figures 1, 2 and 3), the intragroup variation was least among the consulting biostatisticians, although this group had the fewest participants. Regarding intergroup comparisons, the academic clinicians and other stakeholders had roughly similar patterns of rankings of the ethical advantages and disadvantages of ACTs from the participant, researcher, and societal perspectives. The consultant biostatisticians took positions similar to those of the academic clinicians and other stakeholders on the ethical advantages and disadvantages across all scenarios, although their ratings more strongly emphasized the ethical advantages and deemphasized ethical disadvantages. On the other hand, the academic biostatisticians had some overlap with the academic clinicians and other stakeholders, but their anchor points deemphasized ethical advantages and emphasized ethical disadvantages. Over all, intergroup differences were greatest between the academic biostatisticians and the consulting biostatisticians, as they rated oppositely on five of the six ratings, and the tails of their responses on the box plots did not overlap, meaning their ratings differed by more than two standard deviations.

## Discussion

This is the first known empirical study of clinical trial experts’ views on ethical issues in adaptive clinical trials. Previous normative work has debated the ethical construct of clinical trials, and how adaptive clinical trials represent areas where the ethical issues may change based on design features [13,16]. The major concerns raised in these describe a tension between collective ethics (trial validity versus efficiency) and individual ethics (exposure to a better treatment versus fairness of enrolling early versus late in a clinical trial.) A great deal of recent discussion in the clinical trials literature has focused on response adaptive randomization in two-arm trials; however this represents a fairly specific and relatively infrequently used type of ACT [17-22]. Our current investigation builds upon this understanding, and directly examined the opinions of vested stakeholders in the development of ACTs under a special grant from the NIH and FDA to accelerate new discoveries and translate findings into practice [27]. While there were some similar patterns of agreement and disagreement, there were substantive intra-group and inter-group variation. Given that all stakeholders— clinicians, biostatisticians, and others—must work together, understanding the anchor points and values of these groups relative to the potential ethical advantages and disadvantages of ACTS is important for the collaborative efforts needed to make these trials a reality.

### Areas of agreement across stakeholders

Although textual data illustrated many similarities in the understanding of ethical issues of ACTs, the VAS scores demonstrate different anchor points among different stakeholder groups on the relative importance of the ethical advantages and disadvantages of ACTs. The constituent groups agreed that ACTs, including response-adaptive randomization and dropping futile arms, would have ethical advantages for patients. Use of ACTs can help avoid exposing some participants to ineffective treatments, thus offering a clear ethical advantage. For example, the constituent groups agreed that “killing bad drugs” sooner—that is, leveraging the strengths of ACTs to evaluate drugs that do not have potential and ending such trials early—provides ethical advantages. In addition, the ethical advantages of dropping a treatment as soon as possible, and stopping the trial as soon as possible when a treatment is found to be superior, merit highlighting. All stakeholders agreed that adaptations need to be prespecified, and that having a clear understanding of what is being changed or “adapted” is prerequisite for conducting a valid, and hence ethical, ACT.

### Response-adaptive randomization

The point was raised that under response-adaptive randomization, individual participants enrolled early, before any adaptation, would not achieve any additional benefit (or any harm), compared with a trial that lacks response-adaptive randomization or has fixed-interval analysis. However, it is clear that a greater proportion of individuals over the course of a response-adaptive randomized trial would be expected to receive the effective treatment if there ultimately was a more effective treatment being tested. In short, at the population level, participation in an ACT confers an ethical advantage over the course of the trial.

### Unintentional unblinding and biased enrollment

The participant groups identified potential risks and hence potential ethical disadvantages of ACTs. For example, if information about the results of interim analyses were leaked or inferred by clinicians involved in recruiting potential participants, bias could be introduced if the clinician then chose to enroll or not enroll patients on the basis of the information being leaked. For example, in a trial with RAR that is searching for the optimal duration of hypothermia exposure in spinal cord injury, there would be concern for investigator bias if clinician investigators noted that patients at their clinical center/site were being allocated to longer durations of hypothermia. Such risks are greatest if clinicians know what treatment is being assigned, e.g., in an unblinded trial like the spinal cord trial, but also enrollment at their site is relatively high since they won’t necessarily know about enrollment in other sites.

The clinician stakeholders in this study primarily work on multicenter trials in emergency settings where discerning patterns of treatment assignment to different arms of a trial seems unlikely because the actual number of subjects in a given site will be relatively small, even if the trial is unblinded. The concern may be real in open-label trials in which the number of patients in one or more participating centers is large.

### Informed consent

Participants disagreed on whether informed consent would be more complex in ACTs. Existing data suggest that when current informed consent procedures are used, participants consistently fail to grasp the difference between research and treatment, even when there is fixed 1:1 randomization [10]. Thus, the addition of further complexity in an ACT featuring response-adaptive randomization of participants to what appears to be the more effective arm or explaining fixed-interim analysis could appear to increase the complexity of informed consent decisions [31]. Discrepancies in views about this may reflect different value judgments about a “forest versus trees” understanding of the study. If a researcher believes that potential participants must understand the details of the allocation procedure (e.g., response-adaptive randomization) or interim analyses, then the informed consent process could be more difficult. If, alternatively, a “forest” (“big picture”) approach to explanation is followed, then the informed consent process may not provide significantly greater challenges. Among the authors with the most experience in trials using adaptive designs (DB, RL), informed consent has not felt to be substantively more challenging with ACT studies, compared with non-adaptive trials. Future research could explore empirically the difficulty of achieving informed consent in trials that do and do not use adaptive designs. The concept of individual fairness (enrolling early versus late in a ACT), was not substantially explored by this group of stakeholders. Since the ADAPT-IT project focused on neurological emergencies (stroke, spinal cord injury, brain injury following cardiac arrest etc.) and others had substantial oncology experience (cancer patients are unlikely to delay treatment to get a better chance in an ACT) this general concept was not frequently discussed as it is not possible to delay the occurrence of one’s cardiac arrest so as to have a better chance at getting a new treatment in a clinical trial.

### Respondent group variations

Some considerations may help explain the differences between the two biostatistician groups. First, the consultant biostatisticians may have been focusing explicitly on response-adaptive randomization and frequent interim analyses in the context of clear stopping rules, as these elements of high-quality trials are the primary focus of their work. In contrast, the academic biostatisticians may have been considering a broader variety of adaptations and other trial conduct aspects such as maintaining the blind, protocol violations, and implementation of the adaptations. Concerns about the potential biases introduced by unfamiliar types of adaptations may have led academic biostatisticians to generally be more cautious, whereas the consultant groups have more frequently simulated, designed, implemented and analyzed such designs and therefore have less concern about certain forms of potential statistical biases given past experience.

Second, the perspectives on ethical advantages/disadvantages may be highly correlated with experiences in the design and conduct of the more complex adaptive designs. As stated in the FDA guidance, the more complex designs are less understood which may impact the assessment of ethical perspectives. Professional groups, consultant biostatisticians, academic clinicians, academic biostatisticians, and other stakeholders, alike, will make ethical judgments based on their own experiences and professional lenses. For example, consultant biostatisticians bring to the table an approach relying heavily on Bayesian modeling and decision-making. The academic biostatisticians’ views may reflect their experience in the logistical and operational aspects of trials—for example, the complexity of informed consent, protocol deviations and risks for operator bias.

### Study limitations

This study involved a relatively small, but experienced group of clinical trial experts actively developing rigorous clinical trials, although importantly many had little experience with ACT designs. Although this research elicited opinions from various stakeholders regarding the performance of ACT designs, caution should be heeded when extrapolating these opinions to others of the same constituencies or assuming their representativeness in the broader biomedical community. We did not survey institutional review board members, as the ethics review of trials typically occurs after the design phase we focused on, although this would be an interesting area for further study. In addition, medical ethicists would have been able to provide a broader consideration of the ethical issues involved in ACT planning and implementation and would be important to include in future studies. In retrospect, it may have been beneficial for the respondents to consider the ethical aspects of ACTs within the full Emanuel framework (value, scientific validity, fair subject selection, favorable benefit-risk ratio, independent review, informed consent, and respect for the research subjects) [32]. Designers of ACTs may do well to consider these seven elements when comparing a proposed ACT to a more traditional fixed trial in the planning process. In addition, greater involvement of patients or patient advocates is an important part of the trial process and should be included early in the planning of any type of clinical trial [33]. Finally, the expressed opinions on a range of ethical issues come from four constituent groups with widely variable experience and familiarity with ACTs.

## Conclusions

We found substantive differences in views on the ethical aspects of ACTs within and across the key constituencies of consulting statisticians, academic statisticians, academic clinicians, and other stakeholders such as regulators and patient advocates. Collectively, the clinical trial experts participating in this study identified many of the ethically advantageous and potentially challenging aspects of ACTs that are already well known to specialists in this type of trial design. However, at an individual level, perspectives on the ethical advantages and disadvantages of ACTs varied considerably. The four constituencies differ in their weighing of ACT ethical considerations based on their professional vantage points. As a starting point, these data suggest there may be substantive differences in the opinions of key stakeholder constituencies that need to work together to conduct ACTs. Thus, it would seem that data suggest further discussion about the ethics of ACTs is needed to facilitate the moral buy-in that achieves effective teamwork in ACT design and conduct, and ultimately accelerates movement of effective therapies from bench to bedside.

## Additional files

Additional file 1:
**Caption: Visual analog scale survey used for data collection.**
Additional file 2:
**Caption: Mini-focus group discussion guide.**
