# Supplementary material for: Clinical trialist perspectives on the ethics of adaptive clinical trials: a mixed-methods analysis
Source: BMC Med Ethics. 2015 May 3;16:27. doi: 10.1186/s12910-015-0022-z (PMC4424427; doi:10.1186/s12910-015-0022-z)
Supplement: Additional file 1: — Caption: Visual analog scale survey used for data collection. [file 12910_2015_22_MOESM1_ESM.doc]

Date (DD-MMM-YYYY):

|  |  | **-** |  |  |  | **-** |  |  |  |  |
| --- | --- | --- | --- | --- | --- | --- | --- | --- | --- | --- |

|  |  |
| --- | --- |

Subject ID Number:

**PRE-MEETING SURVEY**

For the following statements, please indicate your opinion about the likelihood of each by putting an “X” on the line.

Example:

1. I will go to my dental appointment tomorrow.

|  |  |  |  |  |  |  |  |  | X |
| --- | --- | --- | --- | --- | --- | --- | --- | --- | --- |
| Not at all likely | | Not very likely | | Somewhat likely | | Very likely | | Extremely likely | |

1. NIH grant review panels will **understand** **Phase II** adaptive clinical trial designs.

|  |  |  |  |  |  |  |  |  |  |
| --- | --- | --- | --- | --- | --- | --- | --- | --- | --- |
| Not at all likely | | Not very likely | | Somewhat likely | | Very likely | | Extremely likely | |

Why?___________________________________________________________________________

________________________________________________________________________________

1. NIH grant review panels will **accept as valid** **Phase II** adaptive clinical trial designs.

|  |  |  |  |  |  |  |  |  |  |
| --- | --- | --- | --- | --- | --- | --- | --- | --- | --- |
| Not at all likely | | Not very likely | | Somewhat likely | | Very likely | | Extremely likely | |

Why?___________________________________________________________________________

________________________________________________________________________________

1. NIH grant review panels will **understand** **Phase III** adaptive clinical trial designs.

|  |  |  |  |  |  |  |  |  |  |
| --- | --- | --- | --- | --- | --- | --- | --- | --- | --- |
| Not at all likely | | Not very likely | | Somewhat likely | | Very likely | | Extremely likely | |

Why?___________________________________________________________________________

________________________________________________________________________________

1. NIH grant review panels will **accept as valid Phase III** adaptive clinical trial designs.

|  |  |  |  |  |  |  |  |  |  |
| --- | --- | --- | --- | --- | --- | --- | --- | --- | --- |
| Not at all likely | | Not very likely | | Somewhat likely | | Very likely | | Extremely likely | |

Why?___________________________________________________________________________

________________________________________________________________________________

1. The FDA will **understand** adaptive clinical trial designs relative to regulatory approval.

|  |  |  |  |  |  |  |  |  |  |
| --- | --- | --- | --- | --- | --- | --- | --- | --- | --- |
| Not at all likely | | Not very likely | | Somewhat likely | | Very likely | | Extremely likely | |

Why?___________________________________________________________________________

________________________________________________________________________________

1. The FDA will **accept as valid** adaptive clinical trial designs relative to regulatory approval.

|  |  |  |  |  |  |  |  |  |  |
| --- | --- | --- | --- | --- | --- | --- | --- | --- | --- |
| Not at all likely | | Not very likely | | Somewhat likely | | Very likely | | Extremely likely | |

Why?____________________________________________________________________________

________________________________________________________________________________

1. Researchers will **understand** adaptive clinical trial designs.

|  |  |  |  |  |  |  |  |  |  |
| --- | --- | --- | --- | --- | --- | --- | --- | --- | --- |
| Not at all likely | | Not very likely | | Somewhat likely | | Very likely | | Extremely likely | |

Why?___________________________________________________________________________

________________________________________________________________________________

1. Researchers will **accept as valid** adaptive clinical trial designs.

|  |  |  |  |  |  |  |  |  |  |
| --- | --- | --- | --- | --- | --- | --- | --- | --- | --- |
| Not at all likely | | Not very likely | | Somewhat likely | | Very likely | | Extremely likely | |

Why?___________________________________________________________________________

________________________________________________________________________________

1. Journal peer-reviewers will **understand** adaptive clinical trial designs.

|  |  |  |  |  |  |  |  |  |  |
| --- | --- | --- | --- | --- | --- | --- | --- | --- | --- |
| Not at all likely | | Not very likely | | Somewhat likely | | Very likely | | Extremely likely | |

Why?___________________________________________________________________________

________________________________________________________________________________

1. Journal peer-reviewers will **accept as valid** adaptive clinical trial designs.

|  |  |  |  |  |  |  |  |  |  |
| --- | --- | --- | --- | --- | --- | --- | --- | --- | --- |
| Not at all likely | | Not very likely | | Somewhat likely | | Very likely | | Extremely likely | |

Why?___________________________________________________________________________

________________________________________________________________________________

1. Clinicians will **understand** adaptive clinical trial designs.

|  |  |  |  |  |  |  |  |  |  |
| --- | --- | --- | --- | --- | --- | --- | --- | --- | --- |
| Not at all likely | | Not very likely | | Somewhat likely | | Very likely | | Extremely likely | |

Why?___________________________________________________________________________

________________________________________________________________________________

1. Clinicians will **accept as valid** adaptive clinical trial designs.

|  |  |  |  |  |  |  |  |  |  |
| --- | --- | --- | --- | --- | --- | --- | --- | --- | --- |
| Not at all likely | | Not very likely | | Somewhat likely | | Very likely | | Extremely likely | |

Why?___________________________________________________________________________

________________________________________________________________________________

For the following statements, please indicate the strength of your opinion about
each by putting an “x” along the line.

Example:

1. Liver is my favorite food

X

|  |  |  |  |  |  |  |  |  |  |
| --- | --- | --- | --- | --- | --- | --- | --- | --- | --- |
| Definitely Not | | Probably Not | | Possibly | | Probably | | Definitely | |

1. Adaptive clinical trial designs pose ethical **advantages** from the **patients’** perspective.

|  |  |  |  |  |  |  |  |  |  |
| --- | --- | --- | --- | --- | --- | --- | --- | --- | --- |
| Definitely Not | | Probably Not | | Possibly | | Probably | | Definitely | |

Why?___________________________________________________________________________

________________________________________________________________________________

1. Adaptive clinical trial designs pose ethical **disadvantages** from the **patients’** perspective.

|  |  |  |  |  |  |  |  |  |  |
| --- | --- | --- | --- | --- | --- | --- | --- | --- | --- |
| Definitely Not | | Probably Not | | Possibly | | Probably | | Definitely | |

Why?___________________________________________________________________________

________________________________________________________________________________

1. Adaptive clinical trial designs pose ethical **advantages** from the **researchers’** perspective.

|  |  |  |  |  |  |  |  |  |  |
| --- | --- | --- | --- | --- | --- | --- | --- | --- | --- |
| Definitely Not | | Probably Not | | Possibly | | Probably | | Definitely | |

Why?___________________________________________________________________________

________________________________________________________________________________

16) Adaptive clinical trial designs pose ethical **disadvantages** from the **researchers’** perspective.

|  |  |  |  |  |  |  |  |  |  |
| --- | --- | --- | --- | --- | --- | --- | --- | --- | --- |
| Definitely Not | | Probably Not | | Possibly | | Probably | | Definitely | |

Why?___________________________________________________________________________

________________________________________________________________________________

1. Adaptive clinical trial designs pose ethical **advantages** from the **societal** perspective.

|  |  |  |  |  |  |  |  |  |  |
| --- | --- | --- | --- | --- | --- | --- | --- | --- | --- |
| Definitely Not | | Probably Not | | Possibly | | Probably | | Definitely | |

Why?___________________________________________________________________________

________________________________________________________________________________

18) Adaptive clinical trial designs pose ethical **disadvantages** from the **societal** perspective.

|  |  |  |  |  |  |  |  |  |  |
| --- | --- | --- | --- | --- | --- | --- | --- | --- | --- |
| Definitely Not | | Probably Not | | Possibly | | Probably | | Definitely | |

Why?___________________________________________________________________________

________________________________________________________________________________

1. Adaptive clinical trial designs increase the overall **efficiency** of research.

|  |  |  |  |  |  |  |  |  |  |
| --- | --- | --- | --- | --- | --- | --- | --- | --- | --- |
| Definitely Not | | Probably Not | | Possibly | | Probably | | Definitely | |

Why?___________________________________________________________________________

________________________________________________________________________________

1. Adaptive clinical trials produce **scientifically valid** results.

|  |  |  |  |  |  |  |  |  |  |
| --- | --- | --- | --- | --- | --- | --- | --- | --- | --- |
| Definitely Not | | Probably Not | | Possibly | | Probably | | Definitely | |

Why?___________________________________________________________________________

________________________________________________________________________________

1. Adaptive clinical trials will improve patient care faster than traditional trials.

|  |  |  |  |  |  |  |  |  |  |
| --- | --- | --- | --- | --- | --- | --- | --- | --- | --- |
| Definitely Not | | Probably Not | | Possibly | | Probably | | Definitely | |

Why?___________________________________________________________________________

________________________________________________________________________________

22) Any other comments? (Please use the back of this page if additional space is needed):
