# Supplementary material for: Clinical trialist perspectives on the ethics of adaptive clinical trials: a mixed-methods analysis
Source: BMC Med Ethics. 2015 May 3;16:27. doi: 10.1186/s12910-015-0022-z (PMC4424427; doi:10.1186/s12910-015-0022-z)
Supplement: Additional file 2: — Caption: Mini-focus group discussion guide. [file 12910_2015_22_MOESM2_ESM.doc]

Date (DD-MMM-YYYY):

|  |  | **-** |  |  |  | **-** |  |  |  |  |
| --- | --- | --- | --- | --- | --- | --- | --- | --- | --- | --- |

Subject ID NUMBERs of participants:

Interviewer: _____________________ Interview Location: __________________

**Major questions to answer**

- General impression about adaptive clinical trials
- Experiences with adaptive clinical trials
- Experiences with/views about NIH grant review panels response to adaptive trial designs for phase II trials.
- Experiences with/views about NIH grant review panels response to adaptive trial designs for phase III trials.
- Views about importance of potential early stopping rule penalty in ACT designs.
- Experiences with/views on ethical dimensions, strengths and weaknesses, of adaptive clinical trial designs
- Experience with/views on journal peer review processes to fairly evaluate the strengths and weaknesses of adaptive clinical trials.
- Experiences with/views on adaptive clinical trials compelling enough to change clinical practice.

**Introduction**

Focus group facilitator should introduce self by name and role.

*I am on a research team looking at how researchers develop clinical trials for emergency care. Your opinions are VERY valuable as we try to learn about this. I would like to tape record our conversation so we can be certain not to lose any information you tell me. After the recording is reviewed to check my notes, the recording will be deleted.*

*To assist me in understanding the tapes later, can we go around the table and would each of you introduce yourself?*

**Icebreaker Questions (Note numbers not continuous to match with in person interview questions)**

1. ***Please tell me how long you have been involved in research.***

General perceptions

1. ***Can you tell me your view about what an adaptive trial is?***
2. ***Clarification: general impression of adaptive clinical trials?***
3. ***(If applicable): Tell me about previous experience with adaptive clinical trials***
   1. *What went well?*
   2. *Not so well? (Focus on trials with patients).*
4. ***What about funding? Have you had experience:***
   1. *Submitting yourself ?*
   2. *Reviewing adaptive trial proposals?*

**Funding of ACTs**

1. ***What have been your experiences with how NIH grant review panels have responded to to adaptive trial designs for***
   1. *For phase II trials?*
   2. *For phase III trials?*

*(Probe: If you have not had any experience, what do you think might be their response?)*

1. **I want to explore with you the idea of “PI disincentive for early stopping rule”. One commonly utilized feature of adaptive clinical trials is early looks for futility and efficacy.  If the same question was being asked in a traditional trial with no stopping rules, then the PI would retain funding for the grant and infrastructure for a longer period of time regardless of early signs of lack of efficacy.**
   1. Have you have ever experienced a situation where this could apply?
   2. Do you think that the potential lost funding from an early stopping rule could be a barrier to use of adaptive designs.

**Peer Review / Publication / Dissemination**

1. ***Have you had experience submitting***
   1. *To biomedical journals for publication? Please elaborate.*
   2. *What are major barriers to dissemination?*
   3. *What about reviewing papers with adaptive trials?*
2. ***What has been the response of the journal readership to your published ACTs?***
   1. *Your work?*
   2. *Work of others?*
3. ***Thinking about adaptive designs compared to traditional research designs, how would you compare their respective ability to change clinical practice?*** *<be sure respondents contrast the two>*

**Ethical Dimensions of ACTs. Now I want to explore the ethical dimensions of ACTs.**

1. ***What your thoughts on the ethics of ACTs?***
   1. *Patient /surrogate perspective – advantages? Disadvantages?*
   2. *Researchers’ perspective - advantages? Disadvantages?*
   3. *Societal perspective - advantages? Disadvantages?*
      1. *Considering limited resources [example: trial volunteers and funds for research]*
2. ***Think about informed consent in clinical trials. What is your experience/thoughts related to informed consent in ACTs?***
   1. Patient/surrogate perspective – advantages? Disadvantages?
   2. Researchers’/treating physician perspective - advantages? Disadvantages?
      1. Writing them for IRB
      2. Explaining them to patients
   3. Institutional Review Board (IRB) - advantages? Disadvantages?

(in reviewing the consent process)

1. ***Please think about performance of emergency research under Exception From Informed Consent (EFIC). What are your experiences/thoughts on using ACTS in this context?***

*Probes: Perspective of:*

1. Community consultation
2. Public meetings,
3. How to opt out (eg, use of arm bands)
4. FDA requirement in exception to informed consent

**Regulatory Issues**

1. ***What regulatory issues have you encountered in the incorporation of adaptive designs into clinical trials? (Eg, rigorous Phase III trials)***
   1. FDA perspective?
   2. Local IRB perspective?
   3. IRBs of Collaborating institutions perspective?
2. ***Are there any other issues regarding adaptive clinical trials or clinical trials in general that you think we would be aware of?"***
   1. Perceived barriers?
   2. Strengths?
   3. Logistical hurdles?

**Wrap Up**

This is all the questions I wanted to ask you.

*Is there anything else we did not cover above that you think would be useful for us to know about adaptive clinical trials (either content or your opinions)?*

Thank you very much for participating in this research.

** Interviewer will ask related questions necessary for clarification.*

**Appendix – Ethical Discussion if Needed For Reference**

This brief explanation and scenario can be given if needed to stimulate conversation.

For example, in response adaptive randomization for a placebo controlled trial – the chance of getting placebo versus the active treatment would change as a signal developed that either the placebo or active treatment was working better. This and all changes that occur as the trial goes on are pre-planned and worked out in the trial planning process using numerical simulation and statistics. This type of trial would result in an overall population that had more patients receiving the treatment which is likely to be better. This potentially addresses the “last patient in” paradox. In many clinical trials which ultimately show a difference between treatments, sufficient information existed to suggest that either the new treatment or the control is better at some point earlier than the end of enrollment. Yet, if you are the last patient enrolled into the trial, you have a 50% chance of getting either treatment – despite the existing information that would actually show a difference.
